# *Bacteroides* Microbial Source Tracking Markers Perform Poorly in Predicting *Enterobacteriaceae* and Enteric Pathogen Contamination of Cow Milk Products and Milk-Containing Infant Food

**DOI:** 10.3389/fmicb.2021.778921

**Published:** 2022-01-04

**Authors:** Kevin Tsai, Vivian Hoffmann, Sheillah Simiyu, Oliver Cumming, Glorie Borsay, Kelly K. Baker

**Affiliations:** ^1^Department of Occupational and Environmental Health, University of Iowa, Iowa City, IA, United States; ^2^International Food Policy Research Institute, Washington, DC, United States; ^3^African Population and Health Research Center, Nairobi, Kenya; ^4^Department of Disease Control, London School of Hygiene and Tropical Medicine, London, United Kingdom

**Keywords:** food safety, contamination indicator, *Bacteroides* microbial source tracking markers, *Enterobacteriaceae* indicators, milk source safety, caregiver food hygiene, qPCR

## Abstract

Consumption of microbiologically contaminated food is one of the leading causes of diarrheal diseases. Understanding the source of enteric pathogens in food is important to guide effective interventions. *Enterobacteriaceae* bacterial assays typically used to assess food safety do not shed light on the source. Source-specific *Bacteroides* microbial source tracking (MST) markers have been proposed as alternative indicators for water fecal contamination assessment but have not been evaluated as an alternative fecal indicator in animal-derived foods. This study tested various milk products collected from vendors in urban Kenyan communities and infant foods made with the milk (*n* = 394 pairs) using conventional culture methods and TaqMan qPCR for enteric pathogens and human and bovine-sourced MST markers. Detection profiles of various enteric pathogens and *Bacteroides* MST markers in milk products differed from that of milk-containing infant foods. MST markers were more frequently detected in infant food prepared by caregivers, indicating recent contamination events were more likely to occur during food preparation at home. However, *Bacteroides* MST markers had lower sensitivity in detecting enteric pathogens in food than traditional *Enterobacteriaceae* indicators. *Bacteroides* MST markers tested in this study were not associated with the detection of culturable *Salmonella enterica* and *Shigella sonnei* in milk products or milk-containing infant food. The findings show that while *Bacteroides* MST markers could provide valuable information about how foods become contaminated, they may not be suitable for predicting the origin of the enteric pathogen contamination sources.

## Introduction

Diarrheal diseases remain the third most common cause of preventable illness and death among children under five globally. A significant portion of pediatric diarrheal disease is caused by the ingestion of food that has been contaminated by enteric pathogens transmitted through human and animal fecal matter (Mølbak et al., 1989; Penakalapati et al., 2017). In 2010, 33% of the 351,000 global deaths from foodborne enteric diseases occurred among children under the age of five (WHO, 2015). In developing countries, infant foods are more likely to be stored for prolonged periods and contaminated with bacteria than adult foods (Barrell and Rowland, 1979; Black et al., 1982). Some of this contamination may be caused by unhygienic infant food preparation behaviors that introduce human or animal fecal bacteria, which can then replicate during storage. Before entering the household environment, food products might also be contaminated with enteric pathogens from animal sources during production or from various sources during distribution and sale (Bintsis, 2018; Harris et al., 2018). Traditional water, sanitation, and hygiene (WASH) interventions that aim to reduce diarrhea incidence may have a limited impact on environmental contamination and their ability to prevent or remove enteric pathogens from food remains unclear (Ercumen et al., 2018; Pickering et al., 2019). There is an urgent need to identify where foodborne contamination comes from in the food supply chain preparation and whether WASH interventions would adequately eliminate the risk factors. More evidence on the primary sources of enteric pathogen contamination in infant food in developing countries would help determine whether hygiene interventions targeting the food supply chain versus food preparation provide greater health gains.

Total coliforms and *Enterobacteriaceae*, such as *E. coli*, have been traditionally used as food safety indicator bacteria for contamination from poor hygiene conditions or failures in sanitary food treatment processes. While they are well-validated predictors of food safety, culture-dependent bacterial assays require at least 24 h to generate results. Furthermore, several studies have found that the detection of coliforms or *E. coli* does not correlate with the presence of other enteric pathogens in food (D’AMICO et al., 2008; Jackson et al., 2012; Rangel-Vargas et al., 2015). The detection of total coliforms and *Enterobacteriaceae* does not provide knowledge on the contamination source, as they can thrive in humans and non-human hosts (Sinton et al., 1998; McLellan and Eren, 2014). In addition, many fecal coliform group members such as *Enterobacter, Klebsiella*, and *Citrobacter* bacteria and *E. coli* can persist outside of warm-blooded hosts and multiply in soil and water, especially in tropical climates (Carrillo et al., 1985; Solo-Gabriele et al., 2000; Doyle and Erickson, 2006; Luo et al., 2011). An alternative fecal indicator that reflects recent fecal contamination and distinguishes fecal contamination sources could help public health authorities implement necessary and timely interventions at the appropriate critical control points to mitigate fecal contamination in foods.

*Bacteroides* microbial source tracking (MST) markers have emerged as an alternative water safety indicator. Numerous studies have used human and animal-based *Bacteroides* MST markers to track sources of fecal contamination in recreational water sources and sewages (Stoeckel and Harwood, 2007; Ahmed et al., 2016). The biological properties of *Bacteroides* bacteria that make them valuable indicators of water quality impairment also suggest their potential as alternative indicators to total coliforms and *E. coli* for fecal source tracking of fecal contamination. *Bacteroides* MST markers differentiate between human and non-human fecal contamination by detecting genes from *Bacteroides* that are adapted to living in a specific animal host (McLellan and Eren, 2014). *Bacteroides* also have a short survival time outside of their hosts and do not replicate after they have been released into the environment. Many human and animal-based *Bacteroides* MST markers are able to achieve above 80% sensitivity and specificity when tested in wastewater samples (Ahmed et al., 2016). Field studies using human-sourced BacHum and HF183 MST markers to measure sewage and recreation water contamination found that they did not cross-react with cow feces and found to have sensitivity ranged from 70 to 80% in human and sewage samples (Odagiri et al., 2015; Ahmed et al., 2016; Hughes et al., 2017; Vadde et al., 2019). Validation studies have also shown bovine-sourced BacCow, and BacR markers do not cross-react with human feces, making them helpful in detecting animal-based fecal sources with 100% sensitivity to ruminant feces (Reischer et al., 2006; Odagiri et al., 2015). *Bacteroides* also decay rapidly in freshwater and seawater, with more than 90% of the host-specific *Bacteroides* DNA decaying in 5 days, making them an indicator of recent (vs. persistent) fecal contamination (Bae and Wuertz, 2009). However, there are contrary reports regarding the magnitude and the direction of the correlation between molecular *Bacteroides* MST markers and culture-dependent counts of *E. coli* and *Enterococcus* spp. in water (Ahmed et al., 2016).

Despite being frequently used to track wastewater and recreational water contamination, only a handful of studies have explored the relationship between the detection of enteric pathogens in food and *Bacteroides* MST markers (Ravaliya et al., 2014; Harris et al., 2018; Ordaz et al., 2019). Therefore, evidence on the validity of *Bacteroides* MST markers’ usefulness in identifying and differentiating between environmental and zoonotic sources of fecal bacteria, especially pathogenic bacteria, in food could improve future food safety surveillance programs’ design interventions to prevent foodborne outbreaks.

This study aimed to describe the detection frequency of enteric pathogens, culturable *Enterobacteriaceae* (general *E. coli*, *E. coli* O157: H7*, Shigella sonnei, Salmonella* spp.*, E. aerogene*s, *Proteus mirabilis*) and human-sourced (BacHum, HF183) and bovine-sourced (BacCow, BacR) *Bacteroides* molecular MST markers in vendor milk products and caregiver-prepared milk-based infant foods in peri-urban neighborhoods in Kenya. We also aimed to determine if there are predictive relationships between *Bacteroides* molecular MST markers and enteric pathogens, as detected by bacterial culture and molecular analysis, that perform equally as well as, or better than, standard culture-based bacterial indicators. We hypothesized that the detection of human and bovine-sourced *Bacteroides* MST markers would perform as well or better than *Enterobacteriaceae* culture assays at predicting pathogens detection, thus making *Bacteroides* MST makers a reliable predictor of fecal contamination for raw milk samples and milk-based household infant foods. We also hypothesized that bovine-sourced MST markers would have greater accuracy for pathogens in milk products, reflecting contamination problems that were not effectively removed via treatment. In contrast, we hypothesized that human-sourced MST markers would be more valuable for identifying pathogens introduced in the household, reflecting contamination introduced by the caregiver’s hands. We focus on milk because it has a large consumer base globally and plays a critical role as a primary nutritional source for feeding infants and young children in many parts of the world, including Kenya. Milk is also often a reservoir of foodborne enteric pathogens (Bintsis, 2017). Our lab has shown that milk as an infant food had higher contamination rates than the other popular infant food types and harbored a higher number of enteric pathogen species than other types of food samples, highlighting a need for milk-based food interventions (Tsai et al., 2019). We leveraged an extensive repository of frequently contaminated milk products and infant food samples to improve our statistical power to address study objectives.

## Research Design

### Sampling Location and Sample Collection

Sample collection was completed as part of the joint Safe-Start and Market to Mouth studies in informal settlements of Kisumu, Kenya, from 2018 to 2019, as described elsewhere (Mumma et al., 2019; Hoffmann et al., 2020). The Safe-Start study was approved by the committees from the Great Lakes University of Kisumu (Ref. No. GREC/010/248/2016), the London School of Hygiene and Tropical Medicine (Ref. No. 14695), and the University of Iowa (IRB ID 201804204). Briefly, Safe-Start enumerators performed household visits among caregivers enrolled in the Safe-Start clinical trial at an intermediate point of the trial to observe their food preparation, feeding, and storage behaviors for infant food (Mumma et al., 2019). If caregivers intended to use milk, Market to Mouth enumerators arranged to accompany caregivers to the market to purchase milk for infant food. The enumerators bought the same type and brand of milk, recorded the type of milk, and transported the milk product in a sealed container back to the laboratory in a cooler within 4 h of collection. The milk product was either collected as 250 or 500 ml of sealed packages for packaged milk products, 250 or 500 ml for unpackaged milk products in a previously sterilized metal container, or 50 grams packet for baby formula. The Safe Start enumerators observed caregivers making the food with the milk and collected a sample of the infant food at the point of feeding in a 100 ml Whirlpak bag (Catalog# WPB00679WA, Whirlpak, WI, United States). All the collected samples (*n* = 394 each for milk products and milk-containing infant food) were processed immediately after arriving at the lab.

### Detection of Enteric Bacteria via Pre-enrichment, Selective Bacterial Culture, and Molecular Confirmation

*Salmonella enterica* (*S. enterica*), Enterohemorrhagic *Eschericia coli* O157: H7 (*E. coli* O157: H7), and *Shigella sonnei* were selected for bacterial culturing. All the target bacteria species are common causes of foodborne diarrheal infections and are frequently detected in dairy products (Ahmed and Shimamoto, 2014; Iwu and Okoh, 2019; Tack et al., 2019). Two-step pre-enrichment procedures were used to improve the sensitivity and accuracy of the culture method. To recover and quantify injured or hibernating but viable bacteria without over-replication of the bacteria population, we modified an existing Food and Drug Administration (FDA) protocol involving 24–72 h of warm sample pre-enrichment for 12 h at 4°C. Temperatures lower than 10°C can slow the transition between lag and log phases of replication for most bacteria such that a single live bacterium may undergo replication only a few times during an 18–24 h incubation, making quantification of contamination more accurate (Gibson et al., 1988; Mattick et al., 2003). Specifically, a total of 3 ml or 3 grams of each food sample collected was pre-enriched at 4°C overnight in 3 ml of buffered peptone water (Catalog#76502, Sigma Aldrich, MO, United States) and then incubated at 41°C for 1 h to induce a reproducible state. The pre-enriched samples were membrane-filtered in serial dilution volumes of 1, 0.1, and 0.01 ml on a 0.45 μm membrane sterile filters (Catalog# 13106, Sartorius, Germany) and cultured overnight at 37°C on the MUG *E. coli* O157: H7 selective agar (Catalog# 44782, Sigma Aldrich, MO, United States), which is selective and differential for general *E. coli* and *pathogenic* O157: H7*, Shigella sonnei, S. enterica*, *Enterobacter aerogenes* (or *Klebsiella aerogenes*), and *Proteus* spp. The culture was carried out in duplicate. Negative water controls were also membrane-filtered and cultured daily along with the samples.

Presumptive bacterial pathogen presence for each sample was determined by counting the colony-forming units for each phenotype of interest (*E. coli* O157: H7*, Shigella sonnei, S. enterica*), according to the manufacturer’s protocol for the agar. Up to 5 colonies of each species phenotype were selected using a sterile pipet tip and boiled at 100°C for 5 min in a 1 ml microcentrifuge tube with 100 μl of nuclease-free water to release DNA. If more than one phenotype was cultured from a sample, colony collection and DNA extraction was performed separately for each phenotype of interest observed. Lysate was centrifuged at 12,000 *g* for 5 min and the supernatant containing DNA was transferred to a fresh tube.

Single-plex polymerase chain reaction (PCR) and gel electrophoresis were conducted to confirm presumed *E. coli* O157: H7*, Shigella sonnei, and S. enterica* phenotype isolates. The genes used to screen for the pathogens of interest are the established and species-specific virulence genes that reflect the presence of target pathogens: *rbdE* for *E. coli* O157: H7, *virG*/*ipaH* for *Shigella sonnei,* and *ttr* for *S. enterica* (Supplemental Table 1; Liu et al., 2016a, b). Primers used to detect indicator genes were custom-made by Integrated DNA Technologies (IDT, IA, United States). Positive and negative controls were included in each run. Positive controls for the target genes were obtained by purifying DNA from 10^8 CFU/ml concentrations of reference bacteria strains sourced from BEI (*E. coli* O157: H7: NR-11, *S. enterica*: NR-514, *Shigella sonnei*: NR-519, BEI, VA, United States). For PCR, 2 μl of the DNA template was mixed with 10 μl of the TaqMan Fast Advanced master mix (Catalog# 4444556. Thermo Fisher, MA, United States), 1.6 μl of 5 μM of forward and reverse primer, and 4.8 μl of nucleic acid-free water in a 100 μl microcentrifuge tubes. The PCR cycling was conducted in an Eppendorf thermocycler (Model# 6331. Eppendorf, Germany). The cycling conditions for PCR were: 94°C for 3 min, followed by 40 cycles of 94°C for 30 s, 60°C for 1 min, and 72°C for 1 min, then finished at 72°C for 10 min. The amplified samples underwent gel electrophoresis using the method described by Lee et al. to confirm amplicon presence (Lee et al., 2012). For the gel electrophoresis, 2% agarose gel, TBE running buffer, and ethidium bromide were used as gel, buffer, and DNA dye. Negative water controls were used during PCR and gel electrophoresis to detect issues with background contamination. The PCR and gel electrophoresis run for each sample was completed in duplicates. If the duplicate results did not agree with each other, a third PCR and gel electrophoresis run was conducted to confirm amplicon presence.

For pathogen culture data, a binary variable was created for each sample based upon whether or not it was positive for *E. coli* O157: H7*, Shigella sonnei*, or *S. enterica* after gel-electrophoresis confirmation of the boiled DNA templates. Any *Salmonella* spp. that were *ttr*-negative were considered *S. enterica* negative. Additionally, an overarching *Enterobacteriaceae* indicator presence/absence variable was created if any *Enterobacteriaceae* colonies (general *E. coli*, *E. coli* O157: H7*, Shigella sonnei, Salmonella* spp.*, E. aerogene*s, *Proteus mirabilis*) were observed, regardless of pathogenic identity, to be consistent with food safety monitoring indicators. If the sample was positive for *E. coli* O157: H7*, Shigella sonnei,* or *S. enterica*, the concentration in CFU/ml was calculated for the positive target bacteria via identifying the serial dilution range (1/0.1/0.01) with countable colonies and then standardizing the concentration to cfu per ml or per gram. If the result was too numerous to count for all of the serial dilutions, we assigned the sample as too numerous to count.

### Detection of Enteric Pathogens and MST Markers via Real-Time PCR (qPCR)

When samples were aliquoted for pre-enrichment in the laboratory, another 200 μl/200 mg of each milk source or infant food sample was also aliquoted into a Zymo DNA/RNA Shield tube (Catalog# R1102, Zymo Research, CA, United States) and vortexed. The samples were stored in a − 20°C freezer in Kisumu for subsequent sample transfer and molecular analysis in the United States. Negative water controls were also prepared daily by placing a total of 200 μl of nucleic acid-free water into a separate Zymo DNA/RNA Shield tube. Frozen samples and negative controls were periodically transferred back to Iowa in an ice chest on dry ice. All samples were spiked before extraction with 3 μl of 10^6 unit/μl of live bacteriophage MS2 as a process control to monitor nucleic acid degradation during extraction. The samples were extracted via ZymoBIOMICS™ DNA/RNA extraction mini-kit (Catalog# R2002, Zymo Research, CA, United States) by following the manufacturer’s DNA/RNA co-extraction protocols. Purified DNA/RNA was stored at −80°C.

The enteric pathogen gene targets chosen for food qPCR (Supplemental Table 2) were identical to the previously validated enteric pathogen gene targets chosen for analysis of infant feces in the Safe Start Study and isolates verification above, with the exception of including two human-sourced (BacHum, HF183) and two bovine-sourced (BacR, BacCow) MST markers (Vila et al., 2003; Petri et al., 2008; Clements et al., 2012; Malla et al., 2018). The four selected human and bovine-sourced MST markers were validated by testing their detection frequency and cross-reactivity against the human infant stool samples our lab has collected for the Safe Start study. Human-sourced MST markers were considered as validated if they were detected frequently in human stool samples. Bovine-sourced MST markers were considered validated if they had low or no cross-reactivity in human stool samples.

The extracted samples were tested on a custom-made TaqMan array card (Thermo Fisher, MA, United States) using the Ag-Path-ID One-Step Real-time PCR kit (Catalog# 4387424, Thermo Fisher, MA, United States), and a ViiA7 instrument (Model# AB-ViiA7, Thermo Fisher, MA, United States). For each sample, 40 μl of the DNA/RNA extract was mixed with 50 μl of 2× RT-buffer, 4 μl of 25× AgPath enzyme, 6 μl of nucleic acid-free water, and 0.6 μl of 50 mg/ml bovine serum albumin to prevent amplification inhibition in PCR. The cycling conditions for the TaqMan qPCR runs were: 45°C for 20 min and 95°C for 10 min, followed by 40 cycles of 95°C for 15 s and 60°C for 1 min.

For qPCR analysis, the amplification of a pathogen-specific gene target or a MST marker target was defined as positive for the presence of that pathogen or marker if the cycle-threshold value (Ct value) was less than 35. Any amplification beyond 35 Ct value was classified as negative/extremely low amplification. If positive amplification of pathogen-specific gene or MST marker target was also observed in the negative water control processed on the same day, the sample was classified as negative for that target. For species with two gene targets (Enteroaggregative *E. coli* (EAEC)/ Enteropathogenic *E. coli* (EPEC)/Enterotoxigenic *E. coli* (ETEC)), amplification of either one of the target genes in a given sample would make the sample being classified positive for that particular pathogen type.

### Statistical Analysis

The statistical analysis was completed using Microsoft Excel (Microsoft, WA, United States) and SAS software (Version: 9.4, SAS Institute, NC, United States). A binary indicator (Anypath) was defined as the detection of any one of the target enteric bacteria, viruses, and protozoan pathogen genes. Pathogen diversity (Sumpath) was calculated by adding the total number of the target bacterial, viral, and protozoan enteric pathogens detected by qPCR in a sample. Anypath and Sumpath were tabulated by unpacked, pasteurized, and ultra-heat treated (UHT) “Long-Life” milk source and by type of infant food collected from caregivers. The mean and standard deviation of MS2 Ct value of each MS2 spiked sample and negative control were also calculated to assess the extraction efficiency of the nucleic acid extraction kit.

The detection frequency of Anypath, Sumpath, target enteric pathogens detected by qPCR, culturable *Enterobacteriaceae/S. enterica/Shigella sonnei*/*E. coli* O157: H7, and each validated MST marker was tabulated for milk sources and milk-containing infant food. The sensitivity and specificity between the validated human/non-human MST markers and the 95% confidence interval were calculated and compared to the detection of cultured *Enterobacteriacea* on culture media, the detection of common types of enteric pathogens by qPCR, and the detection of any enteric pathogen by qPCR. Wilcoxon Rank Sum Test was used to determine if the concentration of *E. coli* O157: H7 or *S. enterica* or *Shigella sonnei* detected via culture is associated with MST marker detections.

## Results

### Type of Milk Source and Type of Infant Food Being Prepared by the Caregivers

Of the 394 infant food samples containing purchased milk products, one was dry powdered milk while the remainder contained liquid milk, specifically packaged UHT long-life milk (*n* = 276), followed by packaged fresh pasteurized milk (*n* = 84), and then unpackaged milk (*n* = 33). The most common milk-containing infant food was porridge (*n* = 189), followed by the direct consumption of milk (107 packaged long-life milk, 33 packaged fresh milk, 17 unpackaged milk, one baby formula), and milk-tea (*n* = 35). The remainder of the food was prepared with milk plus cooked/uncooked grain (*n* = 10). Two of the samples collected did not have information on the type of milk-containing infant food that was being prepared.

### Culture Detection of Target Culturable Enteric Bacteria

*Enterobacteriaceae* were isolated more frequently from milk-containing infant foods than milk products (value of *p*<0.01; Table 1). Of the human pathogens in this family, culturable *ttr* positive *S. enterica* was detected more in milk-containing infant foods (6.9%) than milk products (5.1%; value of *p* = 0.13). Among milk products, *S. enterica* was most frequently cultured from unpackaged milk samples. Among milk-containing infant foods, pure milk had a slightly higher detection rate of culturable *S. enterica* than porridge and tea prepared with milk (value of *p* = 0.96). Twenty-four milk products (6.1%) and 66 milk-containing infant foods (16.8%) contained *virG* positive *S. sonnei* (value of *p* = 0.27). None of the samples were *rdb* gene-confirmed *E. coli* O157: H7, although 38.2% milk-containing infant food samples were phenotype positive. Porridge with milk has a higher proportion of containing culturable *S. sonnei* than stored milk and milk tea (value of *p* = 1).

**Table 1:** Detection of any *Enterobacteriaceae,* and of these *Salmonella enterica* and *Shigella sonnei* bacteria in 3 ml or 3 grams of milk products and milk-containing infant food.

	*Enterobacteriaceae* positive % (*n*)	*Salmonella enterica* positive % (*n*)	*Shigella sonnei* positive % (*n*)
Milk products			
Baby formula, *N* = 1	0 (0)	0 (0)	0 (0)
Packaged long life milk, *N* = 276	12.0 (33)	1.8 (5)	0.7 (2)
Packaged fresh milk, *N* = 84	25.0 (21)	2.4 (2)	3.6 (3)
Unpackaged milk, *N* = 33	93.9 (31)	39.4 (13)	57.6 (19)
Overall, *N* = 394	21.6 (85)	5.1 (20)	6.1 (24)
Infant food			
Porridge, *N* = 189	57.7 (109)	5.8 (11)	15.8 (30)
Milk only, *N* = 158	54.4 (86)	8.9 (14)	16.4 (26)
Milk tea, *N* = 35	48.6 (17)	2.9 (1)	17.1 (6)
Uncooked grain, *N* = 9	77.8 (7)	0 (0)	22.2 (2)
Cooked grain, *N* = 1	100 (1)	0 (0)	100 (1)
No record, *N* = 2	100 (2)	50 (1)	50 (1)
Overall, *N* = 394	56.4 (222)	6.9 (27)	16.8 (66)

1 *E. coli* 0157: H7 phenotype not shown due to unconfirmed identity. Other bacteria types not shown due to low frequency (*n* < 5) of detection.

### Evaluation of Nucleic Acid Extraction Kit Performance Using Bacteriophage MS2

The extraction performance of the ZymoBIOMICS™ DNA/RNA extraction mini-kit was evaluated by analyzing the mean and the variance of bacteriophage MS2 across milk source samples, milk-containing infant food samples, and negative control. The low variance in the extrinsic control MS2 Ct value across milk products, infant foods, and negative controls indicated that sample transportation and storage had no impact on DNA and RNA recovery (Supplemental Table 3). The nucleic acid extraction kit we used produced high-quality nucleic acid that was free of PCR inhibition across both samples and controls. Analysis of mean and variance for MS2 by milk source, infant food, and negative water control indicated that MS2 values were similar to each other, which shows that the kit’s extraction efficacy was not affected by the type of food being extracted.

### qPCR Detection of Target Enteric Pathogens

**Figure 1:**
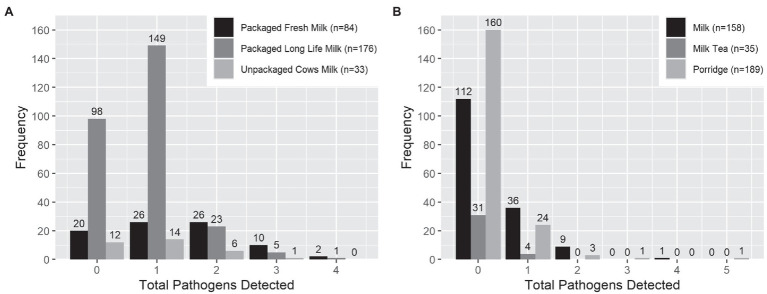
Number of enteric pathogen types detected by qPCR per sample in **(A)** milk products purchased for infant feeding and **(B)** milk-containing infant food prepared by caregivers with those products. *Infant food type with less than 10 samples was excluded from the graph.

Overall and individually, target enteric pathogens were more frequently found by qPCR in milk products than infant food samples (Tables 2 and 3). *S. enterica* was the most common pathogen detected in milk products (54.3%, mean Ct = 33.2, standard deviation = 0.88). However, *S. enterica* was not detected by qPCR in any of the infant foods made from these sources, including the opened containers of pure milk. *Aeromonas* was the most common pathogen detected in infant food (13.5%, mean Ct = 32.8, standard deviation = 1.67), and the second most common in milk products (22.8%, mean Ct = 33.3, standard deviation = 1.22). *Enterotoxigenic E. coli* was the most frequently detected *E. coli* type in both milk products (7.4%, mean Ct = 33.2, standard deviation = 1.40), and infant food (4.3%, mean Ct = 34.1, standard deviation = 0.72). *S. sonnei* was detected at lower frequencies than by culture, with just 0.5% of the milk products and infant foods positive by qPCR compared with 6.3% of the milk products and 16.7% of infant foods positive by culture. Other viral, bacterial, and protozoan pathogens for which we tested occurred in <=3% of samples of infant food (Table 2). Infant food prepared using UHT milk had a lower qPCR enteric pathogen detection rate than the infant food prepared by pasteurized and raw milk (Table 3). Pasteurized milk products were more likely to have multiple types of enteric pathogens detected by qPCR than UHT milk and unpackaged milk. Pure milk was more likely to have multiple enteric pathogens than other milk-containing infant food types (Figure 1).

**Table 2:** Enteric Pathogens Detected by qPCR in DNA and RNA extracted from 0.2 ml or 0.2 gram of milk products and milk-containing infant food.

Pathogen name	Milk products % positive (*n* = 394)	Milk-containing infant food % positive (*n* = 394)
Adenovirus 40–41	0 (0)	0.8 (3)
Adenovirus hexon	0.3 (1)	0.3 (1)
*Aeromonas hydrophila*	22.8 (90)	13.5 (53)
*C. difficile*	0.3 (1)	0 (0)
*C. jejuni*	0 (0)	0 (0)
*Cryptosporidium* 18 s	0 (0)	0.5 (2)
*E. coli* O157: H7	0.5 (2)	0.3 (1)
EAEC*	2.0 (8)	1.3 (5)
EPEC*	3.1 (12)	2.0 (8)
ETEC*	7.4 (29)	4.3 (17)
*Giardia*	0 (0)	2.0 (8)
Norovirus GI	0 (0)	0.3 (1)
Norovirus GII	0 (0)	0 (0)
Rotavirus	0 (0)	0.3 (1)
*Salmonella enterica*	54.3 (214)	0 (0)
*Shigella sonnei*	0.5 (2)	0.5 (2)
*Vibrio cholerae*	0 (0)	0.5 (2)

* *EAEC, Enteroaggregative E. coli; EPEC, Enteropathogenic E. coli; ETEC, Enterotoxigenic E. coli*.

**Table 3:** Comparison of pathogen and microbial source tracking marker detection frequencies in DNA and RNA extracted from 0.2 ml or 0.2 gram of milk products and milk-containing infant food.

	Any target pathogen % (*n*)	BacHum % (*n*)	BacR % (*n*)
Milk products			
Baby formula, *N* = 1	0 (0)	0 (0)	0 (0)
Packaged long life milk, *N* = 276	64.5 (178)	4.0 (11)	0.4 (1)
Packaged fresh milk, *N* = 84	76.2 (64)	1.2 (1)	7.1 (6)
Unpackaged milk, *N* = 33	63.6 (21)	3.0 (1)	15.2 (5)
Overall, *N* = 394	66.8 (263)	3.3 (13)	3.0 (12)
Infant food			
Porridge, *N* = 189	15.3 (29)	15.9 (30)	11.6 (22)
Stored milk, *N* = 158	29.1 (46)	0.0 (0)	3.2 (5)
Milk tea, *N* = 35	11.4 (4)	2.9 (1)	0 (0)
Uncooked grain, *N* = 9	33.3 (3)	22.2 (2)	22.2 (2)
Cooked grain, *N* = 1	0.0 (0)	0.0 (0)	0.0 (0)
No record, *N* = 2	50 (1)	50.0 (1)	0.0 (0)
Overall, *N* = 394	21.1 (83)	8.6 (34)	7.4 (29)

### qPCR Detection of Human and Bovine-Sourced *Bacteroides* MST Markers

For the validation of the target MST markers, among 90 human infant stool samples collected for the Safe Start project, 76.9% were BacHum positive, and 44.4% were HF183 positive. For Bovine-based MST markers, BacR was detected in 11.1% of the human stool samples and BacCow in 38.9% of the stool samples. Overall, *Bacteroides* MST markers were more frequently detected in infant food than milk products. Human-based BacHum (8.6%, mean Ct = 34.3, standard deviation = 1.23) were detected more often than bovine-sourced BacR (7.4%, mean Ct = 34.0, standard deviation = 1.46) in milk-containing infant food, while BacHum (3.3%, mean Ct = 34.4, standard deviation = 0.88) and BacR (3.0%, mean Ct = 34.1, standard deviation = 0.92) had a similar detection rate across all milk products. BacHum was more frequently detected in UHT milk among all of the milk products, while BacR was more frequently detected in unpackaged milk. Of the milk-based infant food types, BacHum and BacR were more frequently to be detected in porridge with milk than pure milk or milk tea, each of which had more than 10 samples available for analysis (Table 3).

### Sensitivity and Specificity of MST Markers Compared to qPCR and Culture-Based Pathogen Detection

**Figure 2:**
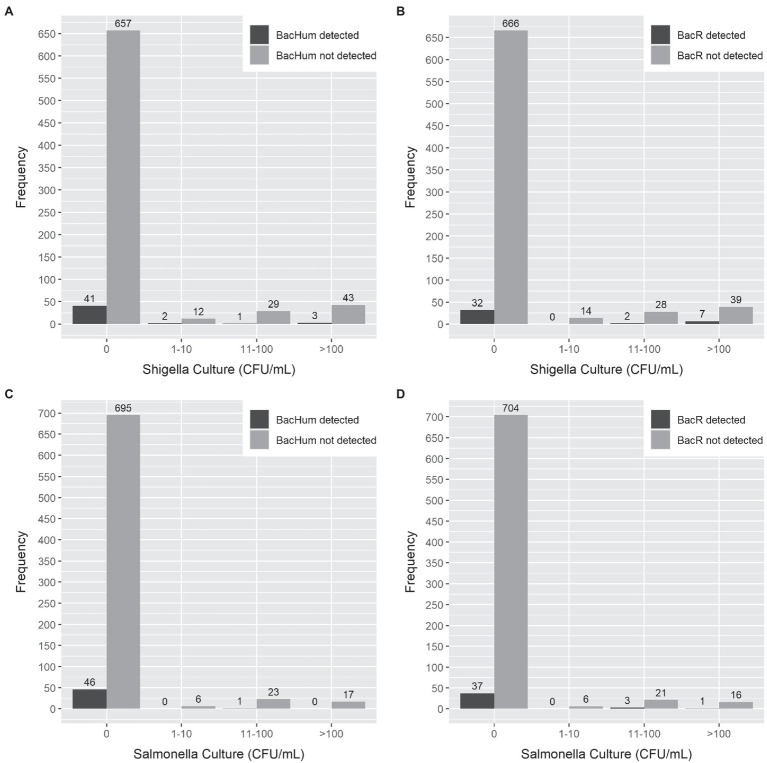
Comparison of MST marker gene detection by qPCR vs. the cell concentration of *Salmonella enterica* and *Shigella sonnei* detection by selective culture in milk products and milk-containing infant food. **(A)** BacHum vs. *Shigella sonnei*. **(B)** BacR vs. *Shigella sonnei*. **(C)** BacHum vs. *Salmonella enterica*. **(D)** BacR vs. *Salmonella enterica*.

Sensitivity and specificity analysis between HF183 and BacCow MST markers and the detection of the enteric pathogen by qPCR or culture was not performed due to statistical limitations of the low detection rate of HF183 and the frequent detection of BacCow in locally collected human stool samples. The sensitivities for BacHum and BacR MST markers for predicting enteric pathogens detected by culture and qPCR were consistently low, while the specificities were moderate to high (Table 4). The *Enterobacteriaceae* culture indicator had better sensitivity but worse specificity for predicting enteric pathogens detected by qPCR compared to the BacHum and BacR MST markers. The concentration of culturable *S. enterica* or *S. sonnei* in a sample was not correlated with the detection of either BacHum or BacR *Bacteroides* MST markers. High concentrations of culturable *S. enterica* or *S. sonnei* were found in samples in which neither BacHum nor BacR *Bacteroides* were detected (Figure 2). Wilcoxon rank-sum tests indicated that the distribution between milk source BacR and *S. enterica* or *S. sonnei* were not equal, but the median for those distributions were zero since most of the BacR positive samples lacked culturable *S. enterica* or *Shigella sonnei* (Table 5).

**Table 4:** Sensitivity and specificity of molecular MST markers and cultured *Enterobacteriaceae* bacteria compared against culture and qPCR detection of *S. enterica* and *S. sonnei* in milk products and milk-containing infant foods.

	Milk product (*n* = 394)		Milk-containing infant food (*n* = 394)	
Any target pathogen (qPCR)				
Indicator	Sensitivity (95% CI)	Specificity (95% CI)	Sensitivity (95% CI)	Specificity (95% CI)
BacHum (Human)	0.02 (0–0.04)	0.95 (0.91–0.99)	0.10 (0.03–0.16)	0.92 (0.89–0.95)
BacR (Bovine)	0.03 (0.01–0.05)	0.97 (0.94–1.00)	0.16 (0.08–0.23)	0.95 (0.92–0.97)
*Enterobacteriaceae* Culture	0.18 (0.13–0.23)	0.71 (0.63–0.79)	0.57 (0.46–0.68)	0.44 (0.38–0.49)
*Salmonella enterica* (Culture)				
Indicator	Sensitivity (95% CI)	Specificity (95% CI)	Sensitivity (95% CI)	Specificity (95% CI)
BacHum (Human)	0 (0–0)	0.97 (0.95–0.98)	0.04 (0–0.11)	0.91 (0.88–0.94)
BacR (Bovine)	0.10 (0–0.23)	0.97 (0.96–0.99)	0.07 (0–0.17)	0.93 (0.90–0.95)
*Enterobacteriaceae* Culture	1 (1–1)	0.83 (0.79–0.86)	1 (1–1)	0.47 (0.42–0.52)
*Shigella sonnei* (Culture)				
Indicator	Sensitivity (95% CI)	Specificity (95% CI)	Sensitivity (95% CI)	Specificity (95% CI)
BacHum (Human)	0 (0–0)	0.96 (0.95–0.98)	0.09 (0.02–0.16)	0.91 (0.88–0.94)
BacR (Bovine)	0.17 (0.02–0.32)	0.98 (0.96–0.99)	0.08 (0.01–0.14)	0.93 (0.90–0.96)
*Enterobacteriaceae* Culture	1 (1–1)	0.84 (0.80–0.87)	1 (1–1)	0.52 (0.47–0.58)
*Salmonella enterica* (qPCR)				
Indicator	Sensitivity (95% CI)	Specificity (95% CI)	Sensitivity (95% CI)	Specificity (95% CI)
BacHum (Human)	0.02 (0–0.04)	0.96 (0.93–0.99)	No qPCR *Salmonella enterica* Detection	
BacR (Bovine)	0.02 (0–0.04)	0.96 (0.93–0.99)		
*Enterobacteriaceae* Culture	0.15 (0.10–0.20)	0.71 (0.64–0.77)		
*Aeromonas* (qPCR)				
Indicator	Sensitivity (95% CI)	Specificity (95% CI)	Sensitivity (95% CI)	Specificity (95% CI)
BacHum (Human)	0 (0–0)	0.96 (0.93–0.98)	0.08 (0–0.14)	0.91 (0.88–0.94)
BacR (Bovine)	0.07 (0.02–0.12)	0.98 (0.97–1)	0.19 (0.08–0.29)	0.94 (0.92–0.97)
*Enterobacteriaceae* Culture	0.22 (0.14–0.31)	0.79 (0.74–0.83)	0.57 (0.43–0.70)	0.44 (0.38–0.49)
ETEC* (qPCR)				
Indicator	Sensitivity (95% CI)	Specificity (95% CI)	Sensitivity (95% CI)	Specificity (95% CI)
BacHum (Human)	0.03 (0–0.10)	0.97 (0.95–0.99)	0 (0–0)	0.91 (0.88–0.94)
BacR (Bovine)	0.03 (0–0.19)	0.97 (0.95–0.98)	0.06 (0–0.17)	0.93 (0.90–0.95)
*Enterobacteriaceae* Culture	0.17 (0.03–0.31)	0.78 (0.74–0.82)	0.47 (0.23–0.70)	0.43 (0.38–0.48)

* ETEC: Enterotoxigenic *E. coli*.

**Table 5:** Median and interquartile concentrations range and Wilcoxon rank-sum test result of *Bacteriodes* MST markers and culturable *Salmonella enterica* and *Shigella sonnei* in milk source and milk-containing infant food.

MST markers (number of positive sample)*	Q1-Q3 (median)	Milk source *Salmonella enterica* value of *p* (W-value)	Q1-Q3 (median)	Milk source *Shigella sonnei* value of p (W-value)
BacHum (13)	0–0 (0)	0.19 (2437.5)	0–0 (0)	0.18 (2411.5)
BacR (12)	0–0 (0)	0.03 (2649)	0–550 (0)	<0.01 (3036)
MST markers (number of positive sample)*		Infant food *Salmonella enterica* value of *p* (W-value)		Infant food *Shigella sonnei* value of *p* (W-value)
BacHum (34)	0–0 (0)	0.17 (6452)	0–0 (0)	0.47 (6750.5)
BacR (29)	0–0 (0)	0.49 (5720)	0–0 (0)	0.45 (5774.5)

* For positive BacHum or BacR only.

## Discussion

This study described the detection frequency for the common food safety indicator *Enterobacteriaceae*, multiple enteric pathogens, and human and bovine *Bacteroides* MST markers in Kenyan milk products and milk-containing infant food through both culture and qPCR approaches. The study also assessed the performance of *Bacteroides* MST markers as an alternative fecal indicator to *Enterobacteriaceae* of enteric pathogen presence in milk based foods. Molecular *Bacteroides* MST markers have been used to assess the source of fecal commination in wastewater and recreational water (Harwood et al., 2014; Ahmed et al., 2016; Korajkic et al., 2018). Their correlation with pathogens is often poor due to the rarity of enteric pathogens in the environment. However, we expected molecular *Bacteroides* MST markers to be consistently detected among enteric pathogen positive foods, and to be at least as sensitive and specific as general *Enterobacteriaceae* indicators. We also expected that the species-specific nature of *Bacteroides* MST markers would result in higher accuracy for bovine-sourced markers predicting pathogens for milk products than the general *Enterobacteriaceae*, reflecting the role of cow upstream pathogen sources in food contamination. Similarly, human-sourced MST markers were expected to have higher accuracy for pathogens in infant food compared to the general *Enterobacteriaceae,* reflecting household members as the most likely contamination source in the household environment. Contrary to expectations, we found no association between the concentration of culturable *S. enterica* and *S. sonnei* and the presence of human and bovine *Bacteroides* MST markers. *Bacteroides* MST markers were frequently absent when enteric pathogens were detected, by both culture and molecular methods, with much lower sensitivity than *Enterobacteriaceae*.

In first examining enteric pathogen detection patterns, we noted that pathogen culture results were in agreement with common knowledge about the bactericidal effects of pasteurization, with *Enterobacteriaceae*, *S. enterica*, and *Shigella sonnei* more frequently detected in unpackaged milk products than packaged milk products. Additionally, the detection of viable *Enterobacteriaceae, S. enterica* and *S. sonnei* was more common in infant food made with these milk products, suggesting that caregivers’ introduced these bacteria to processed milk during food preparation. The qPCR results also provided insights into the contamination profile of milk products and infant weaning food, despite the results not always agreeing with the culture results of selected culturable bacteria and the conventional belief that packaged and treated milk products are safer than unpackaged milk products. Among all milk products, fresh pasteurized milk products had higher qPCR enteric pathogen detection rates than unpackaged and UHT packaged milk products. Inconsistencies between species-specific culture and qPCR assays could be due to PCR detection of non-culturable or non-viable microorganisms that persisted in food through pasteurization, or under detection of viable but non replicating bacteria. We did not employ any sample treatment procedures to determine the viability of pathogens detected by qPCR because some studies suggest viability PCR can lead to false-positive results (Fittipaldi et al., 2012).

Although viability of pathogens cannot be confirmed via molecular gene detection, the prevalence of enteric pathogen genes decreased between purchase of milk products and the point-of-consumption of infant food. This was driven predominantly by a decrease in the detection of *S. enterica* and *Aeromonas* across all milk types of sources from the vendor to the household. This decrease suggested either household food hygiene procedures eliminated some enteric bacteria or residual DNA of dead bacteria in the purchased milk products disintegated. In contrast, the emergence of genes for viruses and protozoans like *Norovirus*, *Rotavirus*, *Cryptosporidium* and *Giardia* in infant food that were not detected in matched milk products reinforces the conclusion of our prior study that food handling practices in the household are an important source of pathogen contamination in infant foods (Hoffmann et al., 2020).

Similar to our molecular and culture pathogen data, both the human and bovine *Bacteroides* MST markers used in this study and *Enterobacteriaceae* were detected more frequently in infant food than milk products. BacHum *Bacteroides* MST markers were detected as frequently as the BacR *Bacteroides* markers in milk products. However, BacR was detected much more frequently in unpackaged milk samples than pasteurized and UHT milk samples, suggesting it may be useful for predicting bovine-based sources of feces and *S. enterica* and *S. sonnei*. Milk could be contaminated by dirt, flies, and/or untreated water originating from unsanitary environments with animal urine and feces (Sampane-Donkor, 2002; Girma et al., 2014). The detection of BacR in a small number of packaged milk products suggested that treatment failures could also be impacting the safety of pasteurized milk. The higher BacHum detection rates in UHT milk likely reflect post-treatment contamination prior to packaging. While BacR trends were similar to bacterial culture trends, *Enterobacteriaceae* detection reflected the same differences across product types and co-occurred with pathogens more consistently.

The higher BacHum and BacR MST marker detection rate in milk-containing infant food than milk products agreed with viral, bacterial, and protozoan pathogen emergence in infant foods implicating caregiver food hygiene behaviors in infant food contamination. Among all milk-containing infant food collected, porridge was more likely to contain *Bacteroides* MST markers than other food types. In Kenya, milk is often added to porridge as part of the diet (Galiè et al., 2021). The milk may not reach a sufficient temperature to kill *Bacteroides* and other microbes if milk is added after boiling, which would explain BacR detection. Another explanation for the differences in MST detection in infant food is that caregivers may handle milk they purchased differently based on whether the milk the caregivers purchased was pasteurized. Therefore, having interventions that target safe infant food preparation by caregivers may reduce the likelihood of *Bacteroides* and/or enteric pathogens in milk-containing foods, especially porridge.

While *Bacteroides* MST markers could provide valuable insights into sources of feces and enteric pathogen contamination in milk and milk-containing foods, they were less sensitive than the conventional *Enterobacteriaceae* indicators in predicting enteric pathogen presence in this large and diverse sample of 786 bovine milk-containing foods. A reliable MST marker should have at least 80% sensitivity and specificity, so to some extent *Enterobacteriaceae* were also not reliable (Harwood et al., 2014). The low sensitivity and specificity between MST markers and enteric pathogens and lack of association between MST markers and the concentration of culturable *S. enterica* and *Shigella sonnei* could reflect that MST markers do not persist in milk products and milk-containing infant food. Although there is limited data on the persistence of *Bacteroides* MST markers in food, *Bacteroides* MST markers’ persistence in recreational waters decreases as water temperature increases (Kreader, 1998; Okabe and Shimazu, 2007). *Bacteroides* MST markers are also less persistent than fecal coliforms and *Enterococcus* in river water at high-temperature (Ballesté and Blanch, 2010). The fact that most milk products and milk-containing infant food are pasteurized or boiled may thus underlie the low detection of *Bacteroides* DNA. Also puzzling is the increased detection of bovine BacR genes in peri-urban Kisumu households where cow ownership is rare, albeit goat, poultry, and companion animal ownership is common (Barnes et al., 2018). We had to exclude *BacCow*, another bovine-base MST marker, from this analysis as it was frequently detected in infant stool samples collected in the Safe Start study. Cross-reactivity of *Bacteroides* MST markers between sources greatly diminishes the value of these markers for differentiation of fecal contamination sources.

There were several limitations to this study. As noted above, quantitative comparison of viable *Bacteriodies* and enteric bacteria between culture and qPCR for this study may have been influenced by culture-dependent and culture-independent methodological gaps. Our culture results may have underestimated actual contamination prevalence if some samples contained low numbers of target bacteria that were not detectable by culture methods after the pre-enrichment procedure. A lack of secondary selective enrichment procedure after the pre-enrichment procedure in the study design could also increase the number of false-negative culturable bacteria detections (Robinson, 2014). We attempted to improve the overall culture detection rate by employing overnight pre-enrichment at refrigeration temperature in peptone water to control replication rates and restricting warm pre-enrichment to 1 h. Overnight refrigeration of food prior to selective culture is not uncommon, and we simply standardized the time interval prior to a limited warm pre-enrichment step. A forthcoming manuscript by our group will describe the overall effectiveness of this protocol versus more extended warm pre-enrichment periods and qPCR-based approaches at recovering and quantifying enteric bacteria from milk. Additionally, the volumes used for molecular assays were about 10x smaller than culture volumes, and the Cq values for detected pathogens and MST markers were near the lower limit of detection. This may have led to false negatives for the qPCR assays as well. In piloting our methods, we sought to filter concentrate bacteria from larger volumes of milk, but milk fat clogged the filters. DNA from larger volumes could have resulted in higher MST and pathogen detection rates and lower Ct/higher concentration values. Future research should seek to improve quantitative protocols for enumerating viable bacterial pathogens in food. This may reduce gaps in accuracy between culture and qPCR methods and improve the assessment of predictive relationships between MST markers and pathogens in food.

Having analytical indicators that could accurately track the source of enteric contaminations could help identify interventions that reduce foodborne exposure to enteric pathogens and the associated disease burden. The preliminary evidence from this study suggests neither of the two *Bacteroides* MST markers investigated were reliable indicators for enteric pathogen contamination of milk products and milk-based food compared with the *Enterobacteriaceae* indicators, regardless of detection method. Although *Bacteroides* MST markers may not be usseful as a complete replacement for *Enterobacteriaceae* indicators, they could still explain the risk factors contributing to recent contaminations in dairy products and infant food. Future research with additional *Bacteroides* MST markers from other animal sources could shed more light on the overall usefulness of *Bacteroides* MST markers in food safety evaluation.

## Data Availability Statement

The raw data supporting the conclusions of this article will be made available by the authors, without undue reservation.

## Ethics Statement

The studies involving human participants were reviewed and approved by Generated Statement: The studies involving human participants were reviewed and approved by Great Lakes University of Kisumu (Ref: GREC/010/248/2016), London School of Hygiene and Tropical Medicine (Ref: 14695), and the University of Iowa (Ref: IRB ID 201804204). Written informed consent to participate in this study was provided by the participants’ legal guardian/next of kin.

## Author Contributions

KT and KB provided the idea design. KT contributed to implementation of the study, data analysis, and manuscript drafting. JM, SS, and GB contributed to the implementation of the study. OC secured the funding for the Safe Start study, while KB and VH secured the funding for Market to Mouth study. All authors contributed to the article and approved the submitted version.

## Funding

This study was supported by the CGIAR Research Program on Agriculture for Nutrition and Health, hosted by International Food Policy Research Institute (IFPRI), and the Dutch Ministry of Foreign Affairs through Netherlands Development Organization under the Voices for Change Partnership. The Safe Start trial, on which the study built, was funded by the United Kingdom Department for International Development through the SHARE Research Consortium.

## Conflict of Interest

The authors declare that the research was conducted in the absence of any commercial or financial relationships that could be construed as a potential conflict of interest.

## Publisher’s Note

All claims expressed in this article are solely those of the authors and do not necessarily represent those of their affiliated organizations, or those of the publisher, the editors and the reviewers. Any product that may be evaluated in this article, or claim that may be made by its manufacturer, is not guaranteed or endorsed by the publisher.
