# Supplementary material for: Bacteroides Microbial Source Tracking Markers Perform Poorly in Predicting Enterobacteriaceae and Enteric Pathogen Contamination of Cow Milk Products and Milk-Containing Infant Food
Source: Front Microbiol. 2022 Jan 4;12:778921. doi: 10.3389/fmicb.2021.778921 (PMC8764403; doi:10.3389/fmicb.2021.778921)
Supplement: Supplementary file 1 [file Table_1.docx]

**Supplemental Tables**

**Supplemental Table 1: Primer and probes of pathogen and MST marker targets for PCR/gel electrophoresis**

| Name of Target | Target | Primer | Sequence | Amplicon Size |
| --- | --- | --- | --- | --- |
| *E. coli* O157: H7 | *rbdE*  [1] | Forward | TTTCACACTTATTGGATGGTCTCAA | 88 bp |
|  |  | Reverse | CGATGAGTTTATCTGCAAGGTGAT |  |
| *Shigella sonnei* | *IpaH*  [1] | Forward | CCTTTTCCGCGTTCCTTGA | 125 bp |
|  |  | Reverse | CGGAATCCGGAGGTATTGC |  |
|  | *virG*  [2] | Forward | TCAGAAAGGTAATTGGCATGGA | 526 bp |
|  |  | Reverse | AGAACCGCGCCCAAAGA |  |
| *Salmonella enterica* | *ttp* [1] | Forward | CTCACCAGGAGATTACAACATGG | 94 bp |
|  |  | Reverse | AGCTCAGACCAAAAGTGACCATC |  |

1. Liu, J., Gratz, J., Amour, C., Nshama, R., Walongo, T., Maro, A., Mduma, E., Platts-Mills, J., Boisen, N., and Nataro, J. (2016a). Optimization of quantitative PCR methods for enteropathogen detection. *PloS One* 11.
2. Liu, J., Platts-Mills, J.A., Juma, J., Kabir, F., Nkeze, J., Okoi, C., Operario, D.J., Uddin, J., Ahmed, S., and Alonso, P.L. (2016b). Use of quantitative molecular diagnostic methods to identify causes of diarrhoea in children: a reanalysis of the GEMS case-control study. *The Lancet* 388**,** 1291-1301.

**Supplemental Table 2: Primer and probes of pathogen and MST marker targets for TaqMan array card qPCR**

| **Custom Assay Name** | **Gene Target** | **Custom Assay Name** | **Gene Target** |
| --- | --- | --- | --- |
| Adenovirus 40-41 [1] | Fiber Gene | *Aeromonas* [1] | Aerolysin |
| *Shigella* [2] | *virG* | *V. cholerae* [1] | *hlyA* |
| Adenovirus [1] | Hexon | Giardia [2] | 18s rRNA |
| Norovirus GI [1] | ORF 1-2 | *C. jejuni-C. coli* [1] | *cadF* |
| Norovirus_GII [1] | ORF 1-2 | *C. difficile* [1] | *tcdB* |
| Rotavirus [1] | NSP3 | *Salmonella. enterica* [1] | *ttr* |
| *Cryptosporidium* spp. [1] | 18s rRNA | Human MST marker 1 [3] | BacHum |
| *C. hominis* [1] | LIB13 | Human MST marker 2 [3] | HF183 |
| *C. parvum* [1] | LIB13 | Bovine MST marker 1 [3] | BacR |
| Enteroaggregative *E. coli* (EAEC) [1] | *Aaic* | Bovine MST marker 2 [3] | BacCow |
| EAEC 2 [1] | *aatA* |  |  |
| Enteropathogenic *E. coli* (EPEC) [1] | *Eae* |  |  |
| EPEC 2 [1] | *Bfpa* |  |  |
| Enterotoxigenic *E. coli* (ETEC) [1] | *LT* |  |  |
| ETEC 2 [1] | *STh_STp* |  |  |
| *E. coli* O157: H7 [1] | *rdbE* |  |  |

1. Liu, J., Gratz, J., Amour, C., Nshama, R., Walongo, T., Maro, A., Mduma, E., Platts-Mills, J., Boisen, N., and Nataro, J. (2016a). Optimization of quantitative PCR methods for enteropathogen detection. *PloS One* 11.
2. Liu, J., Platts-Mills, J.A., Juma, J., Kabir, F., Nkeze, J., Okoi, C., Operario, D.J., Uddin, J., Ahmed, S., and Alonso, P.L. (2016b). Use of quantitative molecular diagnostic methods to identify causes of diarrhoea in children: a reanalysis of the GEMS case-control study. *The Lancet* 388**,** 1291-1301.
3. Malla, B., Ghaju Shrestha, R., Tandukar, S., Bhandari, D., Inoue, D., Sei, K., Tanaka, Y., Sherchand, J., and Haramoto, E. (2018). Validation of host‐specific Bacteroidales quantitative PCR assays and their application to microbial source tracking of drinking water sources in the Kathmandu Valley, Nepal. *Journal of Applied Microbiology* 125**,** 609-619.

**Supplemental Table 3: *MS2* Ct values across household/vendor samples and negative control**

| **Variable** | **N** |  | **Mean** | **Median** | **Std Dev** | |
| --- | --- | --- | --- | --- | --- | --- |
| Household samples | 346 |  | 19.81 | 19.47 | 2.87 |  |
| Vendor Samples | 341 |  | 20.96 | 20.28 | 2.85 |  |
| Negative Water Control | 100 |  | 19.51 | 19.24 | 1.6 |  |
